# Impaired Mitochondrial Bioenergetics Function in Pediatric Chronic Overlapping Pain Conditions with Functional Gastrointestinal Disorders

**DOI:** 10.1155/2021/6627864

**Published:** 2021-08-13

**Authors:** Gisela Chelimsky, Pippa Simpson, Liyun Zhang, Doug Bierer, Steve Komas, Balaraman Kalyanaraman, Thomas Chelimsky

**Affiliations:** ^1^Division of Gastroenterology, Hepatology, and Nutrition, Department of Pediatrics, Center for Pediatric Neurogastroenterology, Motility, and Autonomic Disorders, Medical College of Wisconsin, Milwaukee 53226, WI, USA; ^2^Division of Quantitative Health Sciences, Department of Pediatrics, Medical College of Wisconsin, Milwaukee 53226, WI, USA; ^3^Department of Neurology, Medical College of Wisconsin, Milwaukee 53226, WI, USA; ^4^Department of Biophysics, Medical College of Wisconsin, Milwaukee 53226, WI, USA

## Abstract

**Background:**

Fatigue is often the primary complaint of children with functional gastrointestinal disorders (FGDI) and other chronic overlapping pain disorders (COPC). The basis for this symptom remains unknown. We evaluated mitochondrial function in the white blood cells of these patients.

**Methods:**

This prospective Children's Wisconsin IRB approved study recruited subjects aging 10–18 years from pediatric neurogastroenterology clinics and healthy comparison subjects (HC). Environmental and oxidative stressors can damage the mitochondrial respiratory chain. The known low-grade inflammation in COPC could, therefore, impact the respiratory chain and theoretically account for the disabling fatigue so often voiced by patients. Mitochondrial energy generation can be easily measured in peripheral mononuclear cells (PMC) as a general marker by the Seahorse XF96 Extracellular Flux Analyzer. We measured 5 parameters of oxygen consumption using this methodology: basal respiration (BR), ATP linked oxygen consumption (ATP-LC), maximal oxygen consumption rate (max R), spare respiratory capacity (SRC), and extracellular acidification rate (ECAR), which reflect non-electron chain energy generation through glycolysis. In health, we expect high ATP linked respiration, high reserve capacity, low proton leak, and low non-mitochondrial respiration. In disease, the proton leak typically increases, ATP demand increases, and there is decreased reserve capacity with increased non-mitochondrial respiration. Findings and clinical data were compared to healthy control subjects using a Mann–Whitney test for skewed variables, Fisher's exact test for dichotomous variables, and regression tree for association with functional outcome (functional disability inventory, FDI).

**Results:**

19 HC and 31 COPC showed no statistically significant difference in age. FGID, orthostatic intolerance, migraine, sleep disturbance, and chronic fatigue were present in the majority of COPC subjects. BR, ECAR, and ATP-LC rates were lower in the COPC group. The low BR and ATP-LC suggest that mitochondria are stressed with decreased ability to produce ATP. Tree analysis selected SRC as the best predictor of functional disability: patients with SRC >150 had a greater FDI (more disability) compared to patients with SRC <=150, *p*-value = 0.021.

**Conclusion:**

Subjects with COPC have reduced mitochondrial capacity to produce ATP. Predisposing genetic factors or reversible acquired changes may be responsible. A higher SRC best predicts disability. Since a higher SRC is typically associated with more mitochondrial reserve, the SRC may indicate an underutilized available energy supply related to inactivity, or a “brake” on mitochondrial function. Prospective longitudinal studies can likely discern whether these findings represent deconditioning, true mitochondrial dysfunction, or both.

## 1. Introduction

The term chronic overlapping pain conditions (COPC) emphasizes the cooccurrence of several common chronic pain syndromes without ascribing pathophysiologic assumptions. Previous names for these disorders include functional pain disorders, central sensitization disorders, and medically unexplained syndromes. Several disorders fit this classification, such as migraine headache, functional gastrointestinal disorders (FGID), fibromyalgia, vulvodynia, temporomandibular joint disorder, and interstitial cystitis/bladder pain syndrome [1]. These disorders may also coexist with chronic fatigue and functional autonomic disorders like postural tachycardia syndrome (POTS). These ailments likely involve at least 10–20% of the pediatric population [2–4] and significantly impact daily activity in 40% of those affected [5]. In particular, FGIDs clearly cooccur with other painful disorders such as fibromyalgia, migraine, and chronic fatigue [6]. More importantly, the associated comorbid disorders and their symptoms, not the gastrointestinal issues, drive both the cost and the disability of FGIDs [7, 8].

In adults, fatigue is the third most common comorbidity of irritable bowel syndrome (IBS) [9]. Higher fatigue correlates with greater somatic symptoms (more severe IBS symptoms, larger number of comorbid medical disorders) and psychological comorbidities (anxiety and depression) [9]. Self-rating of health in IBS subjects relates to number of comorbidities and fatigue, not GI symptoms [10]. In turn, morning fatigue correlates with chronic pain [11], and people with chronic fatigue syndrome have lower pain thresholds [12]. Fatigue also generally correlates with psychiatric comorbidities without any implied cause-effect relationship [13].

Despite fatigue's prevalence in so many medical disorders, including COPCs, the pathophysiology of fatigue is unknown. Hypotheses include microinflammation [14, 15] and psychiatry comorbidities [16, 17]. One possibility is that fatigue reflects actual loss of bioenergetic sources, such as reduced ability to generate energy from mitochondria due to oxidative stress from low grade inflammation in the body, as it has been described in FGID [18]. Chacko et al. have developed a Bioenergetic Health index (BHI) based on mitochondrial bioenergetics that reflects the health of the circulating mitochondria in leukocytes or platelets, acting as early instruments to detect metabolic stress in tissues, and therefore, also in the mitochondria [19]. Chacko et al. describe metabolic stress from chronic illness as the combined damage of reactive oxygen species and systemic inflammation on the mitochondria of leukocytes and platelets [19].

In health, based on the BHI, we expect to see high ATP linked respiration, high reserve capacity, low proton leak, and low non-mitochondrial respiration, while, in disease, the proton leak increases, ATP demand increases, and there is decreased reserve capacity with increased non-mitochondrial respiration [19]. Utilizing the Seahorse XF96 Extracellular Flux Analyzer (North Billerica, MA) to assess mitochondrial bioenergetics is not new [20]. Divakaruni et al. wrote an extensive description of the interpretation of the data [21]. In depressed subjects, impaired mitochondrial bioenergetics including routine and uncoupled respiration, spared respiratory capacity (SRC), coupling efficiency, and ATP turnover-related respiration correlated with depressive symptom severity, loss of energy, difficulty concentrating, and fatigue [22]. Extensive work has also been done in cancer to suggest that these findings truly reflect the state of the mitochondria [23, 24]. These manifestations clearly recapitulate the severe fatigue and “brain fog” seen in 90% of pediatric FGID patients [25]. The aim of this study was to determine if, similar to the findings in depression [22], mitochondrial bioenergetics in children with COPC are reduced.

## 2. Methods

**Figure 1:**
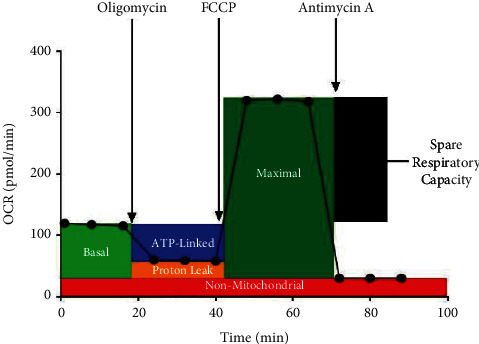
Schematic description of the Seahorse analysis. FCCP = carbonyl cyanide-4-phenyl-hydrazone; OCR = oxygen consumption rate.

This prospective Children's Hospital of Wisconsin IRB approved study recruited subjects between June 2015 and May of 2018 in children aged 10–18 years from the pediatric neurogastroenterology and autonomic disorders clinic. Blood was obtained from youth with COPC and in carefully screened healthy comparison group (HC) with no functional disorder or chronic medical condition of any type. The comparison group was enrolled based on advertisement without aiming at matching for age or gender. The Oxygen Consumption Rate (OCR) and Extracellular Acidification Rate (ECAR) measurements were obtained utilizing the Seahorse XF96 Extracellular Flux Analyzer (North Billerica, MA). Peripheral Blood Mononuclear Cells (PMBCs) were seeded in a PS V7 cell culture plate at a density of 3.25 × 10^5^ cells per well in a assay media supplemented with 1 mM pyruvate and 80 uL of RPMI, centrifuged for 10 min, and then placed in a non-CO_2_ incubator for 1 hr @37C prior to analysis to avoid any temperature variances on the plate that could affect experimental results, followed by prompt analyzed using the Seahorse XF96 Extracellular Flux Analyzer. Oligomycin (1 ug/ml), carbonyl cyanide-4-phenyl-hydrazone (FCCP) (1 uM), and a cocktail of 1 uM Antimycin A and 1 uM rotenone to block complex 3 and 1, respectively, were added to properly determine mitochondrial and non-mitochondrial function. We measured OCR-linked basal respiration (BR), ATP linked oxygen consumption rate (ATP-LC), maximal respiration oxygen consumption rate (max R), non-mitochondrial linked oxygen consumption rate, and spare respiratory capacity (SRC) (Figure 1).

We collected the following data from a systematic chart review: (1) demographics including age and sex and medical history; (2) comorbid symptoms including symptoms of chronic fatigue (>6 months), dizziness, syncope >3 times/life, GI symptoms with functional GI diagnoses based on fulfillment of Rome IV criteria, migraine headaches as per the 2013 International Classification of Headaches Disorders [26] and fibromyalgia assessed by tender-point score [27], pelvic pain, bladder pain syndrome, chronic regional pain syndrome (CRPS), and temporomandibular joint disorder (TMJ). We also recorded any history of posttraumatic stress disorder (PTSD) and panic disorder. To assess the clinical state of the subjects, we utilized a functional questionnaire, the Functional Disability Inventory (FDI) [28], thought to be an excellent measure of overall clinical status and a better assessment than simple pain reports. The FDI measures daily functions on a 5-point Likert scale such as ambulation, recreation, capacity for social interaction, performing chores, going to school, sleep, and eating. Validated for children with chronic abdominal pain, it shows excellent test-retest reliability within 2 weeks, and moderate at 3 months. It correlates well with school-related disabilities, and both the child-report and the parent-report scores correlate with abdominal pain measures and other somatic symptoms [29]. The FDI was only obtained in the COPC group, not in the HC.

Continuous variables are reported as median and interquartile range (IQR) and categorical variables as *n* (%). To compare differences between groups, a Mann–Whitney test was used for continuous, and a Fisher's exact test for categorical variables. Spearman correlation test was used to examine the correlations among the mitochondrial bioenergetic variables, and their correlations with FDI as well. To investigate the relationship of age, gender, BR, ATP-LC, max R, SRC, and ECAR with functional status as reflected in the FDI, a regression tree, optimized by least absolute deviation with 10% leave out samples for cross validation, was performed using Salford systems CART. Significance was attributed with a two-sided *p* value <0.05. All other data analyses were performed using SAS 9.4.

## 3. Results

Of 74 patients enrolled, 24 were excluded due to lack of mitochondrial bioenergetic data (assay malfunctioning or too few cells to run assay). To perform the Seahorse analysis, we need a total of 3.25 × 10^5 PBMCs when performing the final resuspension. We found many samples with an insufficient concentration of PBMCs, which could have been due to loss of cells through sample handling and isolation of PBMCs. This is similar to a standard clinical assay returning a result of “quantity not sufficient”.

Of the remaining 50 subjects, 19 were HC and 31 had a COPC. Neither age (HC: 15.1 (14.3–16.1) yrs; COPC 16.5 (14.6–17.7) yrs; *p*=0.06) nor gender differed (HC 16 (84%) females; COPC 22 (71%) females; *p*=0.33) between the groups. The clinical characteristics of the COPC group included an FGID in 22 (71%) with chronic idiopathic nausea in 14 (48%), cyclic vomiting syndrome (CVS) 5 (17%), irritable bowel syndrome (IBS) (not subclassified, but the vast majority are IBS-C) 17 (57%), functional abdominal pain NOS 13 (45%), and functional dyspepsia 11 (38%). Extra-gastrointestinal comorbidities included orthostatic intolerance in 26 (90%), migraine in 21 (72%), chronic fatigue in 13 (43%), syncope in 11 (38%), TMJ in 6 (21%), fibromyalgia in 6 (21%), complex regional pain in 3 (10%), interstitial cystitis/painful bladder syndrome in 2 (7%), and myofascial pelvic pain in 1 (3%). In relation to the psychiatric comorbidities, 4 (14%) reported a panic disorder, and 6 (21%) reported PTSD.

**Figure 2:**
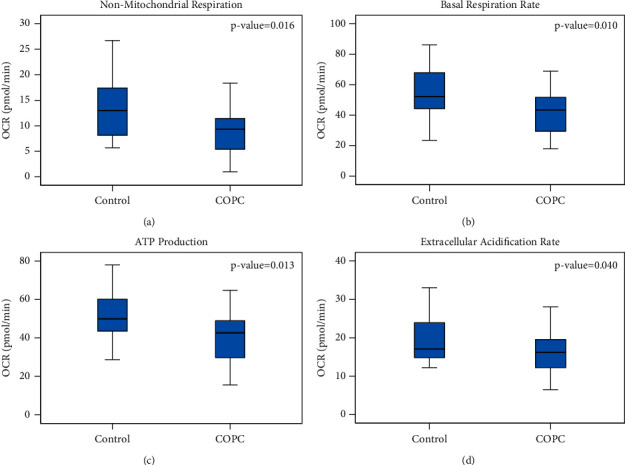
Oxygen consumption rate in non-mitochondrial respiration, basal respiration rate, ATP production (ATP-LC), and extracellular acidification rate (ECAR) in healthy controls and COPC.

Non-mitochondrial linked oxygen consumption rate, BR, ATP-LC, and ECAR were significantly lower in the COPC group (Table 1 and Figure 2) compared to HC. SRC statistically was not different in the 2 groups (*p* > 0.28).

In both COPC and HC groups, BR correlated with SRC, ATP-LC, and MaxR (Table 2). We investigated whether the severity of the patient's clinical syndrome in the COPC group, as reflected in our best measure, the FDI, might correlate with each of the respiratory impairments. No correlation emerged between FDI and BR (*r* = 0.002, *p* > 0.99), MaxR (*r* = 0.19, *p*=0.34), and ATP production (*p* = ATP-LC (*r* = -0.01, *p*=0.95), nor SRC (*r* = 0.22, *p*=0.25) and ECAR (*r* = 0.22, *p*=0.26). Interestingly, ECAR showed a trend for correlation with SRC (*r* = 0.46, *p*=0.06) and ATP-LC (*r* = 0.41, *p*=0.08) in the HC group, while other correlations were stronger in the COPC group (Table 2).

Within the COPC group, females had a higher SRC than males (*p*=0.048).

In a tree analysis, a possible threshold of 150 for SRC was found. Patients who had SRC >150 has a higher FDI than others with SRC <=150, *p*-value = 0.021.

## 4. Discussion

The main finding in this study is that youth with COPC have a reduced mitochondrial bioenergetic capacity compared to healthy control subjects, carrying important clinical and pathophysiologic implications. Clinically, these children and adolescents are often considered as having school avoidance and psychiatric issues and sometimes treated as having no physiologic basis for their complaints. We do not know if this finding demonstrates a basis for the frequent complaint of severe fatigue associated with these disorders, or whether it might simply reflect deconditioning, but it certainly merits further investigation.

Since we did not know if any putative mitochondrial defect might be inherited or acquired, to further understand the role of mitochondria in COPC, we examined the function of the mitochondria rather than looking for a structural mutation by looking at mitochondrial bioenergetics utilizing PMBC. Chacko et al. [19] reported a Bioenergetic Health Index (BHI). A healthy individual should have high ATP production (ATP-LC), high SRC, low non-mitochondrial OCR, and low proton leak. Under increasing oxidative stress, these parameters reverse [19]. Blood leukocytes, as well as platelets, are exposed to circulating factors associated with metabolic stress. Since these cells contain mitochondria, they may provide a functional marker in diseases where inflammation or metabolic stress might play a significant role, such as the COPCs migraine, irritable bowel syndrome, and fibromyalgia [30–32].

It is unclear whether the present finding of reduced bioenergetic capacity reflects a consequence of a predisposition for a COPC or simply deconditioning. A predisposition might reflect genetic or epigenetic changes. In contrast, a consequence could reflect a change in mitochondrial control or modulation rather than any genetic process. For example, it is known that cardiac myocyte mitochondrial function is strongly modulated by the vagus [33]. Whether a similar modulation might impact peripheral blood monocytes is unknown. Since the majority of subjects with COPC demonstrate low vagal modulation [34–36], we believe an acquired (and, therefore, potentially reversible) mechanism may be more likely. This in turn might explain why mitochondrial studies focusing on genetic etiologies have been somewhat inconclusive. Further studies of bioenergetic capability once subjects with COPC improve clinically will help better define whether the observed deficits are reversible, and thus likely acquired.

While the most affected functions were basal respiration and ATP linked oxygen consumption, which depend on the respiratory chain itself, non-mitochondrial energy generation and extra-cellular acidification rates, neither of which depend on the respiratory chain, were also affected for reasons that are unclear (Figure 2). However, these findings favor an acquired defect over an inherited defect, which would have had a more specific impact. Interestingly, in contrast to the BHI index described by Chacko et al., our COPC subjects showed lower non-mitochondrial oxygen consumption, a marker of health [19]. It is not clear why the tree selected a higher spare respiratory capacity as the best predictor of higher functional disability. A higher spare respiratory capacity is generally thought to be healthy [19]. However, in the COPC population, it is possible that higher spare capacity results from less actual energy generation (lower BR in the COPC group) or, perhaps more likely, that the mitochondria in the cells in COPC are not as affected as in patients with dysfunctional bioenergetics such as in diabetes or cardiovascular diseases [19].

Abnormal mitochondrial bioenergetic function also occurs in some psychiatric disorders. Findings nearly identical to those in this study were present in patients with depression. The severity of bioenergetic dysfunction correlated with clinical fatigue [37]. In veterans with PTSD, mtDNA copy numbers are decreased, unrelated to any associated Major Depressive Disorder [38]. Supporting the hypothesis that fatigue most closely reflects the finding of abnormal mitochondrial bioenergetics, higher ventricular cerebrospinal fluid lactate measured by proton magnetic resonance spectroscopy imaging in chronic fatigue syndrome subjects correlated directly with their fatigue level [39]. Future work will need to include a direct assessment of fatigue through existing validated questionnaires in our population to determine if a similar relationship holds.

The exploratory nature of this study directly leads to many of its limitations. Not assessing lactate in our subjects reduced the richness of the interpretation of the anaerobic glycolysis findings. Second, since the FDI did not correlate with the mitochondrial bioenergetics, a measure of fatigue may more accurately reflect mitochondrial bioenergetics. Given that abnormal mitochondrial bioenergetics are present in psychiatric conditions, future studies need to include structured evaluation of PTSD, depression, and anxiety. In this study, we have only had formal records for PTSD. Furthermore, the finding of abnormal bioenergetics may not be specific to COPC. Future studies will require a “sick” comparison group with another chronic disorder of a different type (e.g., Crohn's or other inflammatory disorder) to determine the specificity of these findings to COPC vs. just “being sick” and will benefit from a measure of deconditioning such as maximal oxygen uptake.

In conclusion, subjects with COPC have decreased mitochondrial bioenergetics when compared to healthy age matched controls. The mechanism of the bioenergetic impairment could involve predisposing genetic factors or acquired changes that reverse as disease improves. Prospective longitudinal studies compared to other nonpainful chronic disorders will distinguish these possibilities.

## Figures and Tables

**Table 1:** Comparison in mitochondrial bioenergetic between healthy controls and youth with chronic overlapping pain conditions.

(pmol/min)	HC, median (IQR) *n* = 19	COPC, median (IQR) *n* = 31	*p*-value
Non mitochondrial respiration	13.0 (7.9, 18.0)	9.3 (5.3, 11.7)	0.016
BR	52.3 (44.2, 69.7)	43.4 (27.1, 51.8)	0.010
SRC	117.8 (100.3, 209.7)	118.33 (69.4, 161.1)	0.281
ATP production	49.9 (43.2, 65.4)	42.7 (27.3, 50.2)	0.013
ECAR	17.1 (14.7, 24.0)	16.2 (12.1, 19.5)	0.040

ECAR = extracellular acidification rate; OCR = oxygen consumption rate; BR = basal respiratory rate; SRC = spare respiratory capacity. All mitochondrial bioenergetics measurements are in pmol/min; HC = healthy controls; COPC = chronic overlapping pain conditions; IQR = interquartile range.

**Table 2:** Correlation across bioenergetic variables in COPC and HC.

Correlation	COPC	HC
BR with SRC	*R* = 0.76, *p* < 0.0001	*R* = 0.89, *p* < 0.0001
BR with ATP-LC	*R* = 0.98, *p* < 0.0001	*R* = 0.99, *p* < 0.0001
BR with maximal	*R* = 0.83, *p* < 0.0001	*R* = 0.93, *p* < 0.0001
BR with ECAR	*R* = 0.69, *p* < 0.0001	*R* = 0.46, *p*=0.06
ECAR with SRC	*R* = 0.72, *p* < 0.0001	*R* = 0.51, *p*=0.025
ECAR with ATP-LC	*R* = 0.68, *p* < 0.0001	*R* = 0.41, *p*=0.08

## Data Availability

The deidentified data may be available by request after obtaining the appropriate approval of our IRB.

## Abbreviations

BR: Basal respiration
COPC: Chronic overlapping pain conditions
ECAR: Extracellular acidification rate
FDI: Functional disability inventory
FGID: Functional gastrointestinal disorders
HC: Healthy comparison group
IBS: Irritable bowel syndrome
OCR: Oxygen consumption rate
PMBC: Peripheral blood mononuclear cells
POTS: Postural tachycardia syndrome
PTSD: Post traumatic stress disorder
SRC: Spared respiratory capacity.
